# A New Model of Development of the Mammalian Ovary and Follicles

**DOI:** 10.1371/journal.pone.0055578

**Published:** 2013-02-07

**Authors:** Katja Hummitzsch, Helen F. Irving-Rodgers, Nicholas Hatzirodos, Wendy Bonner, Laetitia Sabatier, Dieter P. Reinhardt, Yoshikazu Sado, Yoshifumi Ninomiya, Dagmar Wilhelm, Raymond J. Rodgers

**Affiliations:** 1 Research Centre for Reproductive Health, Discipline of Obstetrics and Gynaecology, School of Paediatrics and Reproductive Health, Robinson Institute, University of Adelaide, SA, Australia; 2 Institute of Health and Biomedical Innovation, Queensland University of Technology, Kelvin Grove, QLD, Australia; 3 Faculty of Medicine, Department of Anatomy and Cell Biology, McGill University, Montreal, QC, Canada; 4 Faculty of Dentistry, McGill University, Montreal, QC Canada; 5 Division of Immunology, Shigei Medical Research Institute, Okayama, Japan; 6 Department of Molecular Biology and Biochemistry, Okayama University Medical School, Okayama, Japan; 7 Department of Anatomy and Developmental Biology, Monash University, Clayton, VIC, Australia; Montana State University, United States of America

## Abstract

Ovarian follicular granulosa cells surround and nurture oocytes, and produce sex steroid hormones. It is believed that during development the ovarian surface epithelial cells penetrate into the ovary and develop into granulosa cells when associating with oogonia to form follicles. Using bovine fetal ovaries (n = 80) we identified a novel cell type, termed GREL for Gonadal Ridge Epithelial-Like. Using 26 markers for GREL and other cells and extracellular matrix we conducted immunohistochemistry and electron microscopy and chronologically tracked all somatic cell types during development. Before 70 days of gestation the gonadal ridge/ovarian primordium is formed by proliferation of GREL cells at the surface epithelium of the mesonephros. Primordial germ cells (PGCs) migrate into the ovarian primordium. After 70 days, stroma from the underlying mesonephros begins to penetrate the primordium, partitioning the developing ovary into irregularly-shaped ovigerous cords composed of GREL cells and PGCs/oogonia. Importantly we identified that the cords are always separated from the stroma by a basal lamina. Around 130 days of gestation the stroma expands laterally below the outermost layers of GREL cells forming a sub-epithelial basal lamina and establishing an epithelial-stromal interface. It is at this stage that a mature surface epithelium develops from the GREL cells on the surface of the ovary primordium. Expansion of the stroma continues to partition the ovigerous cords into smaller groups of cells eventually forming follicles containing an oogonium/oocyte surrounded by GREL cells, which become granulosa cells, all enclosed by a basal lamina. Thus in contrast to the prevailing theory, the ovarian surface epithelial cells do not penetrate into the ovary to form the granulosa cells of follicles, instead ovarian surface epithelial cells and granulosa cells have a common precursor, the GREL cell.

## Introduction

Knowing how the fetal ovary develops is important particularly for human medical conditions such as premature ovarian failure and polycystic ovary syndrome (PCOS). PCOS is the most common endocrine condition affecting an estimated 5–7% of women of reproductive age in Western societies, and is characterised by hyperandrogenemia, hirsutism, chronic anovulation and polycystic ovaries [1]. Recent evidence suggests that predisposition to PCOS occurs in the developing fetal ovary, specifically affecting the development of the stromal compartments [2]. The other major condition affected by development of the ovary is premature ovarian failure which could be due to a poor endowment of follicles which are formed during fetal development of the ovary [3].

Knowledge of some of the key events of the developing ovary has been established [4], [5], particularly the behaviour of germ cells. It is known that the primordial germ cells (PGCs) arise from the yolk sac and migrate under the control of stem cell factor through the primitive gut into dorsal mesentery and then laterally to the gonadal ridges. These ridges develop on the abdominal side of the mesonephros that operates as a functional kidney in the mammalian fetus until the metanephros assumes this role. On arrival at the developing XX genital ridges the primordial germ cells proliferate as oogonia and subsequently enter meiosis, unlike germ cells in the developing testis. The proliferating oogonia in association with somatic cells are partitioned into irregularly-shaped ovigerous cords radially-orientated towards and open to the surface of the ovary. Later in development, commencing at the base of the cords, the somatic cells closely associate with oogonia and together develop into primordial follicles. The oogonia enlarge and develop into oocytes and the somatic cells develop into the follicular epithelial granulosa cells. Many of the molecular regulators of these events, particularly for the germ cells, have been identified [6]. However, knowledge of the origins and lineages of somatic cells and of the events of regionalization of the ovary into the tunica albuginea, cortex and medulla are not universally agreed upon.

Granulosa cells were originally considered to be derived from the mesonephros and more recently from the ovarian surface epithelium (reviewed recently [4], [5]). The mesonephros is a complex structure with many different cell types, including stromal cells, endothelial cells and different epithelia associated with its nephrons. In mammals the mesonephros is a transient organ during fetal development. However, in females it contributes tubules to the hilus and medulla of the ovary, and these persist into adulthood and are referred to as the rete ovarii. The evidence that these structures give rise to granulosa cells came from early observations that rete ovarii can have a close association with oocytes [7], [8]. This was further strengthened by evidence that the presence of rete ovarii was important for follicle formation [9]. More recent publications suggest that the ovarian surface epithelial cells penetrate into the ovary and associate with germ cells to form the granulosa cells of follicles [10], [11]. There is little information or discussion on the origin of the ovarian surface epithelium as most would have presumed that it expanded from the original surface of the mesonephros and gonadal ridge. In addition the different specialized stromal compartments have been inadequately examined.

In the current study we used bovine ovaries because the adult ovary has defined regions with a medulla, cortex and tunica in proportions similar to the human ovary [12]. Using markers of different cell types and of extracellular matrix found in stroma and basal laminas, we chronologically tracked the fate of somatic cells during ovarian development and conclude that the granulosa cells and ovarian surface epithelial cells arise from a common precursor cell, the Gonadal Ridge Epithelial-Like (GREL) cell.

## Materials and Methods

### Tissues

Fetuses of pregnant *Bos taurus* cows were collected at T&R Pastoral abattoir at Murray Bridge, SA, with their permission, and then transported on ice to the laboratory. Crown-rump length was measured to estimate gestational age [13]. All ovaries were either fixed in situ or frozen in O.C.T. compound (ProSciTech, Thuringowa, QLD, Australia) for immunohistochemistry (n = 53) or used for cell culture (n = 27).

### Gender Determination

To confirm the gender of young fetuses (smaller than 8 cm), genomic DNA was extracted from the tail samples using the Wizard® SV Genomic DNA Purification System (Promega Australia, Alexandria, NSW, Australia) according to the manufacturer’s instructions. Genomic DNA was amplified with a primer pair (sense primer: 5′-TCACTCCTGCAAAAGGAGCA-3′, antisense primer: 5′-TTATTGTGGCCCAGGCTTG-3′), specific for a region in the SRY-determining sequence, and primers specific for the 18S ribosomal RNA gene sequence [14] in separate reactions. SRY product sequences were verified by automated sequencing (3730 DNA analyser; Applied Biosystems, Mulgrave, VIC, Australia).

### Cell Culture

Fetal ovaries (size range 5–92 cm, corresponding age estimated as 67–268 days) were cut into small pieces and treated with 1 mg/ml collagenase type I (GIBCO, Carlsbad, CA, USA) in Hank’s balance-salt solution with Ca^2+^ and Mg^2+^ (Sigma-Aldrich, St Louis, MO, USA) for 1 h at 39°C. Cells were resuspended in DMEM/F12 medium containing 5% fetal calf serum, 50 U/ml penicillin (Sigma-Aldrich), 50 µg/ml streptomycin sulfate (Sigma-Aldrich), 0.25 µg/ml fungizone (GIBCO) and seeded into 24-well plates (Nunc A/S, Roskilde, Denmark) and incubated at 39°C in 5% CO_2_ in air. The culture medium was changed after 24 h.

### Cell Culture for Microarray

Cells from dispersed fetal ovaries (procedure described above) were seeded in a 6-well plate and incubated at 39°C in 5% CO_2_ in air, and cultured until the cells were 70% confluent. Two fetal GREL cell cultures (31 and 32 cm crown-rump-length/corresponding age of 127 and 130 days of fetal development) were selected and compared with fibroblastic cells isolated from the medullar stroma of an adult bovine ovary by treatment with 5 mg/ml collagenase type I (GIBCO, Carlsbad, CA, USA) in Hank’s balance-salt solution with Ca^2+^ and Mg^2+^ (Sigma-Aldrich, St Louis, MO, USA) for 1 h at 39°C and subsequent culture in DMEM/F12 medium containing 5% fetal calf serum, 50 U/ml penicillin (Sigma-Aldrich), 50 µg/ml streptomycin sulfate (Sigma-Aldrich), 0.25 µg/ml fungizone (GIBCO) at 39°C in 5% CO_2_ in air. All cultures were harvested with 800 µl Trizol (Invitrogen Pty Ltd, Mulgrave, VIC, Australia) and frozen at −80°C.

### Microarray

Total RNA was extracted from cultured cells using Trizol according to the manufacturer’s instructions (Invitrogen). Following confirmation of the quality of the RNA and cDNA synthesis, hybridizations to GeneChip Bovine Genome Arrays (Affymetrix, Santa Clara, CA, USA) and scanning were performed according to Affymetrix protocols at the Adelaide Microarray Facility (Adelaide, SA, Australia) with a Genchip 3′ IVT Express kit (Affymetrix). First-strand cDNA synthesis on 250 ng of total RNA for each sample was performed using a T7-linked oligo-dT primer, followed by second strand synthesis. Biotinylated cRNA was generated from the cDNA by *in vitro* transcription and hybridized to Bovine Genome Affy arrays. The arrays were then washed and stained with streptavidin-phycoerythrin (final concentration 10 µg/ml). Signal amplification was achieved by using a biotinylated anti-streptavidin antibody. The array was then scanned according to the manufacturer’s instructions. Any inter- or intra-array variation due to technique was minimised by background subtraction with the Robust Multi Array (RMA) algorithm and quantile normalization with log_2_ transformation. All samples passed all quality controls during analysis. Differences in gene expression between the different cultured cells compared in our arrays were determined by one way ANOVA with Benjamini-Hochberg post hoc False Discovery Rate (FDR) tests for multiple comparisons. The fold change in gene expression was determined from the non log transformed signal data after correction and normalization.

### Histology

Tissues (n = 8) were fixed in 4% paraformaldehyde (Merck Pty Ltd, Kilsyth, VIC, Australia) in 0.1 M phosphate buffer (pH 7.4) and then embedded in paraffin by standard methods. Sections (3 µm) were cut using a CM1850 V2.2 Leica microtome (Leica Microsystems, Nussloch, Germany) for subsequent hematoxylin-eosin staining and immunohistochemistry [15]. Sections of O.C.T.-embedded whole frozen ovaries (n = 30) were cut at 10 µm using a CM1800 Leica cryostat (Leica Microsystems), mounted on Superfrost glass slides and stored at –20°C until used for hematoxylin-eosin staining and immunohistochemistry [15].

### Immunohistochemistry

Antigens were localized by using an indirect immunofluorescence method as described previously [16]. Table 1 summarizes the antibodies used and relevant fixation conditions. Sections were also treated with 4′,6′-diamidino-2-phenylindole dihydrochloride (DAPI) solution (Molecular Probes, Eugene, OR, USA) to identify cell nuclei. Negative controls included no primary antiserum and non-immune mouse, rabbit, rat or goat sera. No staining of fetal ovaries was observed with these controls.

**Table 1:** Primary antibodies, secondary antibodies and labeling, and fixation conditions used for immunohistochemistry and the number of ovaries examined for each antigen.

Antigen (species)	Host Species, Code/Clone Number, Epitope Sequence, Source, Concentration or Dilution, Fixation	Secondary antibody* (Catalogue Number)/Conjugated Fluorophores (Catalogue Number)	Number of Ovaries Examined
**OCT3/4 (human)**	goat, sc-8628/N-19, N-terminus, Santa Cruz^a^,2 µg/ml paraffin/1 µg/ml frozen, 10% BFS	FITC-conjugated AffiniPure donkey anti-goat(705-095-147) or biotin-SP-conjugated AffiniPure donkey anti-goat IgG (705-065-147)/Cy3-conjugated streptavidin (016-160-084)	21
**DAZL (mouse)**	rabbit, ab34139, synthetic peptide conjugated to KLHfrom within residues 250 to C-terminus, Abcam^b^, 1.25 µg/ml, 10% BFS	biotin-SP-conjugated AffiniPuredonkey anti-rabbit IgG (711-066-152)/Cy3-conjugated streptavidin	12
**VASA (human)**	rabbit, ab13840, synthetic peptide conjugated to KLHfrom within residues 700 to C-terminus, Abcam^b^,2.5 µg/ml, 10% BFS or 100% acetone	FITC-conjugated AffiniPure donkey anti-rabbit(711-096-152)	25
**FOXL2 (human)**	rabbit, none, CMMASYPEPEDTAGTLL/CWDHDSKTGALHSRLDL, Dagmar Wilhelm, 1∶100, 10% BFS	biotin-SP-conjugated AffiniPure donkey anti-rabbit IgG/Cy3-conjugated streptavidin	15
**COUP-TFII (human)**	mouse, H7147, 43–64 aa, Perseus Proteomics^c^, 10 µg/ml,10% BFS	biotin-SP-conjugated AffiniPure donkey anti-mouse IgG (715-066-151)/Cy3-conjugated streptavidin or Cy3-conjugated AffiniPure donkey anti-mouse IgG (715-166-151)	5
**SF1 (human)**	rabbit, ab103679, 70–100 amino acids, Abcam^b^, 1∶50, 10% BFS	biotin-SP-conjugated AffiniPure donkey anti-rabbitIgG/Cy3-conjugated streptavidin	13
**Laminin 111 (mouse)**	rabbit, L9393, from basement membrane of EHS mousetumor, Sigma^d^, 10 µg/ml paraffin/5 µg/ml frozen, 10% BFS	Cy3-conjugated AffiniPure donkey anti-rabbitIgG (711-166-152) or FITC-conjugated AffiniPure donkey anti-rabbit (711-096-152)	37
**Collagen type I (bovine)**	mouse, ab6308/COL-1, full length protein, Abcam^b^, 7 µg/ml,100% ethanol	Cy3-conjugated AffiniPure donkey anti-mouse IgG (715-166-151)	26
**Collagen type IV (mouse)**	rabbit, ab6568, full length protein, Abcam^b^, 2.75 µg/ml,10% BFS	biotin-SP-conjugated AffiniPure donkey anti-rabbit IgG/Cy3-conjugated streptavidin	25
**Collagen type IV α1 (human)**	rat, none, C-terminus, [63], 1∶100, 100% acetone	Cy3-conjugated AffiniPure donkey anti-rat IgG (712-166-150)	27
**Collagen type XVIII (mouse)**	rabbit, #92462, peptide between non-collagenousdomain 11 at C-terminus, [64], 1∶300, 100% ethanol	biotin-SP-conjugated AffiniPure donkey anti-rabbit IgG/Fluorescein/DTAF-conjugated streptavidin (016-010-084)	24
**Perlecan (mouse)**	rat, MAB1948/A7L6, heparin sulphate proteoglycan fromEHS mouse tumor, Millipore^e^, 10 µg/ml, 10% BFS or 100%acetone for combination with von Willebrand factor	biotin-SP-conjugated AffiniPure donkey anti-rat IgG (712-066-153)/Cy3-conjugated streptavidin or FITC-conjugated AffiniPure donkey anti-rat (712-096-153)	20
**Nidogen 1 (mouse)**	rabbit, ab14511, recombinant full length protein, Abcam^b^, 1∶200, 10% BFS	biotin-SP-conjugated AffiniPure donkey anti-rabbit IgG/Cy3-conjugated streptavidin or Cy3-conjugated AffiniPure donkey anti-rabbit IgG	13
**Nidogen 2 (mouse)**	rabbit, ab14513, recombinant full length protein, Abcam^b^, 1∶200, 10% BFS	biotin-SP-conjugated AffiniPure donkey anti-rabbit IgG/Cy3-conjugated streptavidin	13
**Fibrillin 1 (bovine)**	mouse, MAB1919/11C1.3, zonular fibrils, Millipore^e^, 5 µg/ml, 10% BFS	FITC-conjugated AffiniPure donkey anti-mouse (715-096-151)	11
**Fibrillin 3C (human)**	rabbit, 4307-1, C-terminal half, [65], 1∶50, 10% BFS	biotin-SP-conjugated AffiniPure donkey anti-rabbit IgG/Fluorescein/DTAF-conjugated streptavidin	24
**Versican (human)**	mouse, 12C5, hyaluronan binding region, HybridomaBank Iowa^f^, 3.5 µg/ml, 10% BFS	biotin-SP-conjugated AffiniPure donkey anti-mouse IgG/Fluorescein/DTAF-conjugated streptavidin	13
**Decorin (bovine)**	mouse, DS1, nd, Hybridoma Bank Iowa^f^, 3.8 µg/ml, 10% BFS	biotin-SP-conjugated AffiniPure donkey anti-mouse IgG/Fluorescein/DTAF-conjugated streptavidin	12
**Fibronectin (bovine)**	rabbit, AB2047, fibronectin extracted and purified from plasma, Millipore^e^, 1∶80, 100% acetone	biotin-SP-conjugated AffiniPure donkey anti-rabbit IgG/Fluorescein/DTAF-conjugated streptavidin	11
**Plakophilin-2 (human)**	mouse, Pkp2-518, 527–72 amino acids, PROGEN^h^, 1∶200,10% BFS	biotin-SP-conjugated AffiniPure donkey anti-mouse IgG/Cy3-conjugated streptavidin	10
**Desmoglein-2 (human)**	rabbit, ab85632, 72–121 amino acids, Abcam^b^, 5 µg/ml,10% BFS	biotin-SP-conjugated AffiniPure donkey anti-rabbitIgG/Cy3-conjugated streptavidin	10
**Cytokeratin 18 (human)**	mouse, C8541/CY-90, nd, Sigma^d^, 1∶1000, 10% BFS	biotin-SP-conjugated AffiniPure donkey anti-mouse IgG/Fluorescein/DTAF-conjugated streptavidin	25
**Cytokeratin 19 (human)**	mouse, nd, nd, Boehringer-Mannheim^i^, 0.25 µg/ml, 10% BFS	biotin-SP-conjugated AffiniPure donkey anti-mouse IgG/Fluorescein/DTAF-conjugated streptavidin	15
**Biotinylated lectin**	*Bandeiraea simplicifolia*, L3759, nd, Sigma^d^, 1∶200, 10% BFS	Fluorescein/DTAF-conjugated streptavidin	9
**von Willebrand** **factor (human)**	rabbit, IS527, full length protein isolated from plasma, DAKO^k^, 1∶1600, 100% acetone	biotin-SP-conjugated AffiniPure donkey anti-rabbitIgG/Cy3-conjugated streptavidin	10
**Ki67 (human)**	mouse, M7240/MIB-1, nd, DAKO^k^, 1∶3200, 10% BFS	FITC-conjugated AffiniPure donkey anti-mouse	27

nd - not determined,

a distributed by ThermoFisher Scientific Inc, Scoresby, VIC, Australia;

b Sapphire Bioscience Pty Ltd, Waterloo, NSW, Australia;

c Perseus Proteomics Inc, Tokyo, Japan;

d Sigma Chemical Co, St Louis, MO, USA;

e Millipore Australia Pty Ltd, Kilsyth, VIC, Australia;

f Developmental Studies Hybridoma Bank, Iowa City, IA, USA;

g Invitrogen Australia Pty Ltd, Mulgrave, VIC, Australia;

h PROGEN Biotechnik GmbH, Heidelberg, Germany;

i Boehringer Ingelheim GmbH, Ingelheim am Rhein, Germany;

j distributed by Immuno Pty Ltd, St Peters, NSW, Australia;

k Dako Australia Pty Ltd, Botany, NSW, Australia,

* All secondary antibodies and fluorophores were from Jackson ImmunoResearch Laboratories Inc. (West Grove, PA, USA) and used 1∶100.

### Electron Microscopy

For epoxy embedding, tissues (n = 15) were fixed in modified Karnovsky’s fixative (2.5% glutaraldehyde, 2% paraformaldehyde in 0.1 M phosphate buffer) at 4°C overnight, and processed for both light and electron microscopic examination as reported previously [17].

## Results

### Characterization of the Developing Ovary

Monolayer cultures of dispersed cells from all stages of bovine fetal ovaries examined (67–268 days) contained areas of spindle-shaped fibroblastic cells, and areas of polygonal-shaped cells (shown in Fig. S1) subsequently identified as GREL cells. Cells with a granulosa cell phenotype were also present when cells were grown from older fetuses. We performed microarray analysis of two clonally enriched GREL cell isolates. A selected list of genes which were more than 3 fold up-regulated in fetal GREL cells with respect to adult ovary fibroblasts as determined by microarray analysis is shown in Table 2. The complete data sets are uploaded to the GEO database under the accession number GSE42838. The log_2_ hybridization intensity for each gene and the fold changes are also presented (Table 2). Some of the genes expressed in GREL cells, such as plakophilin, desmoplakin, StAR (steroidogenic acute regulatory protein) and cytokeratin 19, have been identified before as expressed in the adult human ovarian surface epithelium [18], [19], [20]. However, other ovarian surface epithelium markers, such as adipophilin, fibulin 2, merocin or mucin 1 [18], were not expressed in the GREL cells and, additionally, these cells did not require culture conditions that are needed for culture of ovarian surface epithelium (i.e. media containing 10 or 15% of bovine fetal serum and the culture has to remain undisturbed for at least 3–4 days to allow cell attachment [19], [20]), suggesting that these GREL cells were not ovarian surface epithelial cells, but correspond to the GREL cells. Based on the results of the microarray analyses, antibodies against cytokeratin 19, plakophilin-2 and desmoglein-2 were chosen to locate these cells in tissue sections. Whilst these molecules are not specific to GREL cells, they did produce a staining pattern of GREL cells different to that of true surface epithelial cells as illustrated and discussed below.

**Table 2:** A list of differentially expressed genes in two independent GREL cell isolates (1 and 2 from fetuses of 31 and 32 cm crown-rump-length/corresponding age of 127 and 130 days of fetal development), compared with fibroblast cells isolated from adult bovine ovaries.

Gene Symbol	Gene Name	RefSeq Transcript ID	Intensity*			Fold Changes	
			Fibroblast	GREL 1	GREL 2	GREL 1 vs Fibroblast	GREL 2 vs Fibroblast
**KRT19**	Keratin 19	NM_001015600	7.1	11.3	11.6	18.6	22.8
**S100A2**	S100 calcium bindingprotein A2	NM_001034367	8.6	10.5	10.7	3.9	4.5
**ANXA3**	Annexin A3	NM_001035325	5.5	8.5	8.9	7.5	10.3
**NELL2**	NEL-like 2 (chicken)	NM_001102084	4.6	8.6	8.5	16.1	15.2
**ALDH1A2**	Aldehyde dehydrogenase 1 family, member A2	XM_615062	4.8	8.6	8.5	13.5	12.5
**DSG2**	Desmoglein 2	XM_584890	2.3	6.9	8.3	24.1	61.3
**CXCR7**	Chemokine (C-X-C motif) receptor 7	NM_001098381	5.5	8.2	8.2	6.5	6.2
**SMS**	Spermine synthase	NM_001035471	5.9	8.0	8.1	4.3	4.7
**CA3**	Carbonic anhydrase III, muscle specific	NM_001034437	4.6	7.0	7.4	5.3	7.0
**STAR**	Steroidogenic acute regulatory protein	NM_174189	3.5	8.0	7.0	22.1	11.1
**PKP2**	Plakophilin 2	NM_001083729	3.3	6.4	6.0	8.7	6.5
**PODXL**	Podocalyxin-like	XM_868371	2.5	6.5	6.0	15.9	11.3
**DSP**	Desmoplakin	XM_592197	3.0	6.5	5.9	11.8	7.7
**LSR**	Lipolysis stimulated lipoprotein receptor	NM_001083394	3.3	5.7	5.7	5.2	5.2
**TSPAN2**	Tetraspanin 2	NM_001034657	2.0	5.3	4.9	9.9	7.6
**KRT7**	Keratin 7	NM_001046411	2.3	4.4	4.9	4.1	5.9
**EFNA5**	Ephrin-A5	NM_001076432	1.7	3.9	4.5	4.3	6.7
**GJB2**	Gap junction protein, beta 2, 26 kDa	NM_001083637	1.4	4.0	3.3	5.9	3.7

* Values for signal intensity and subsequent fold change were generated in Partek Genomics Suite (Partek Inc, St Louis, MO, USA) and the full sets of data can be found in the GEO database under the accession number GSE42838.

Using markers for germ cell differentiation, and GREL, stromal and endothelial cells as well as markers for the basal lamina components and stromal cell matrices (Tables 3 and 4) we characterized developmental changes of fetal ovaries chronologically from the gonadal ridge stage to the mature ovarian phenotype. Table 1 also lists the number of ovaries immunostained for each of the 26 antibodies used.

**Table 3:** Characterization of cell types (++ strongly expressed,+expressed, (+) weakly expressed, +/− inconsistently expressed, − not expressed).

	Primordial Germ Cells	Oogonia	Early Oocytes	Follicular Oocytes	GREL Cells	Granulosa Cells	Surface Epithelium	Stromal Cells
**OCT3/4**	+	+/−	−	+/−	−	−	−	−
**DAZL**	−	+	−	−	−	−	−	−
**VASA**	−	+/−	+	+	−	−	−	−
**FOXL2**	−	−	−	−	+	+	+/−	(+)
**Cytokeratin 18**	−	−	−	−	+	+/−	++	−
**Cytokeratin 19**	−	−	−	−	+–++	+	++	−
**COUP-TFII**	−	−	−	−	−	−	−	+
**Plakophilin-2**	−	+/−	+/−	−	+	+	++	−
**Desmoglein-2**	−	+/−	+/−	−	+	+	++	−

**Table 4:** Expression of extracellular matrix molecules in various ovarian structures during development (++ strongly expressed/thick fibres,+expressed, (+) weakly expressed/thin fibres, - not expressed).

	Basal Lamina				Stromal Matrix			
Extracellular Matrix Molecule	Ovigerous Cords	Follicle	Ovarian Surface	Sub-Endothelial	Between Cords	Tunica Abuginea	Cortex	Medulla
**Laminin 111**	+	+	+	+	(+)	+	−	−
**Collagen type I**	−	−	−	−	++	++	++	++
**Collagen type IV**	+	+	+	+	+	+	−	−
**Collagen type XVIII**	+	+	+	+	−	−	−	−
**Perlecan**	+	+	+	+	+	+	−	−
**Nidogen 1**	+	+	+	+	(+)	(+)	(+)	(+)
**Nidogen 2**	+	+	+	+	(+)	+	(+)	(+)
**Fibrillin 1**	−	−	−	−	++	++	++	++
**Fibrillin 3**	−	−	−	−	+	−	(+)	−
**Fibronectin**	−	−	−	−	++	++	++	+
**Decorin**	−	−	−	−	++	++	++	++
**Versican**	−	−	−	−	+	(+)	(+)	−

#### The early stage ovary (<70 days)

**Figure 1:**
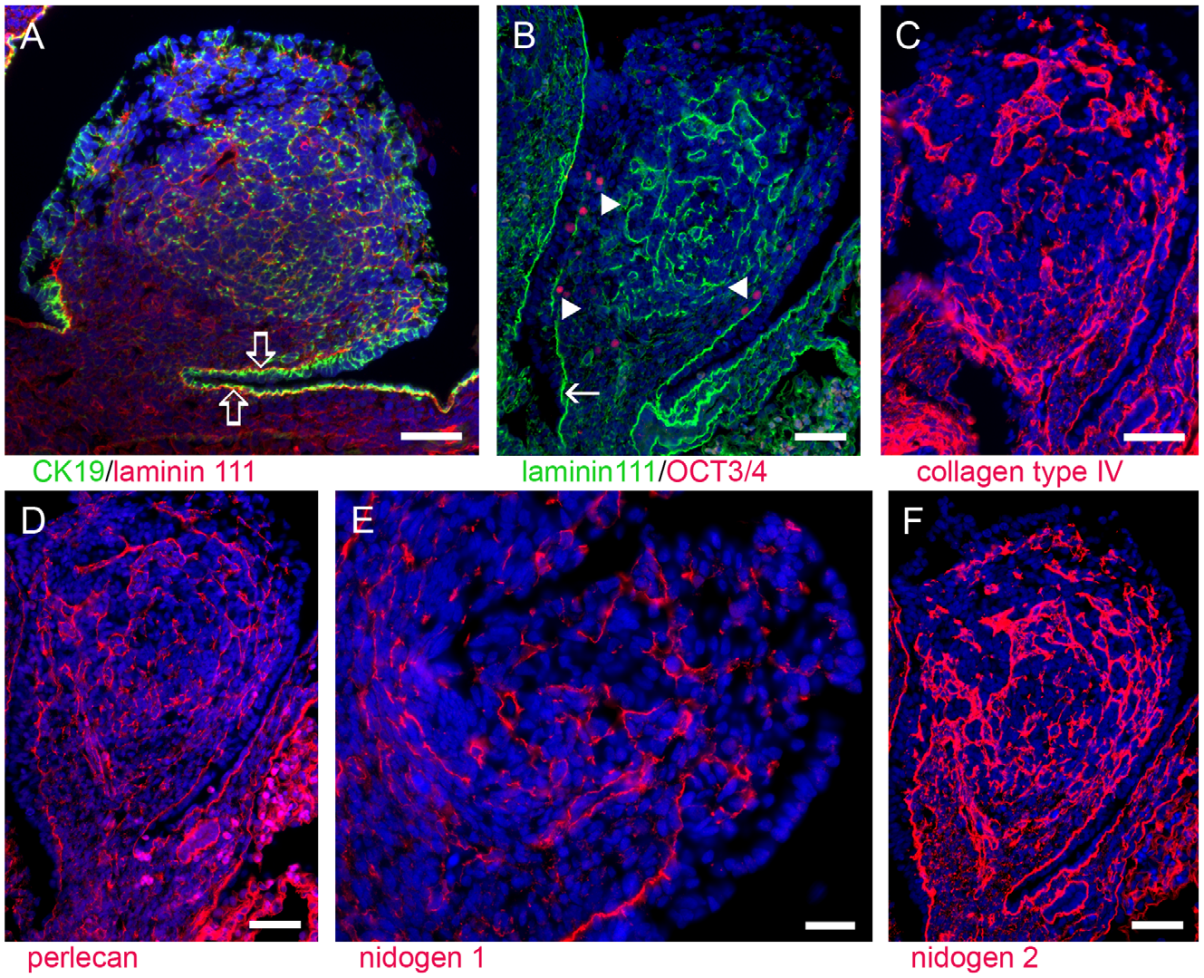
The early-stage ovary. At this stage the ovary was composed of mostly GREL cells, expressing cytokeratin (CK) 19 (A, green), interspersed with OCT3/4 positive PGCs (B, red, arrowheads). The ovary did not have a defined surface epithelium except at its base where it arose and protruded from the mesonephros and where it was strongly stained for cytokeratin 19 (A, green, open arrows) and was under laid by a basal lamina containing components of laminin 111 (A, red and B, green, arrows), collagen type IV (C, red), perlecan (D, red) and nidogens 1 (E, red) and 2 (F, red). Basal lamina material was interspersed throughout the ovary. Nidogen 2 and collagen type IV were more strongly expressed than components of laminin 111, nidogen 1 and perlecan, even in the cytoplasm of some cell clusters. Nuclei were counterstained with DAPI (blue). Gestational age: 63 days. Bars: A–D, F = 50 µm; E = 25 µm.

**Figure 2:**
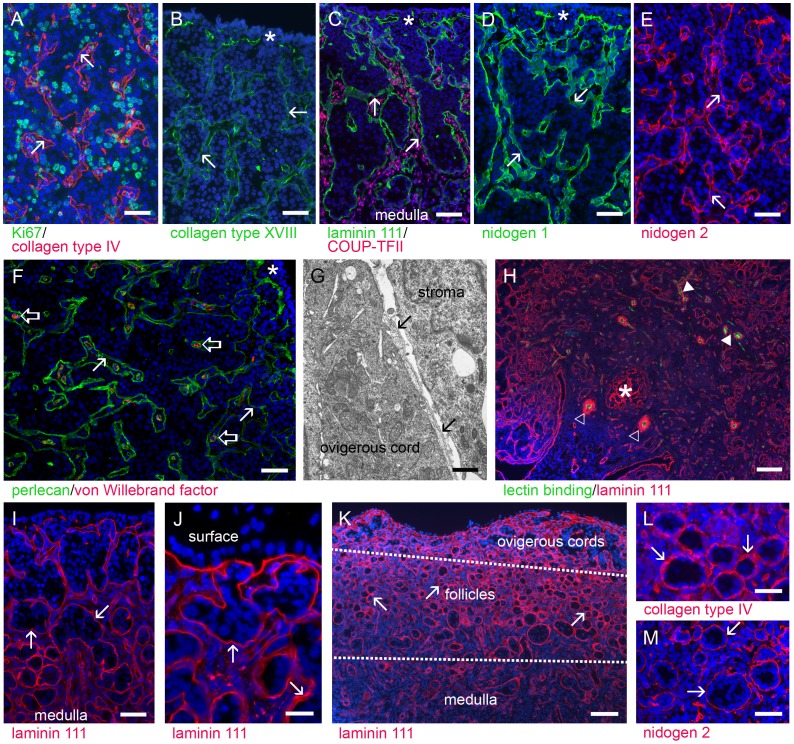
The ovarian cortex is rich in ovigerous cords separated from the penetrating stroma by a basal lamina. The basal lamina (marked with arrows) contains collagens type IV (A, red) and type XVIII (B, green), components of laminin 111 (C, green and H, red), nidogens 1 (D, green) and 2 (E, red) and perlecan (F, green). The electron micrograph shows that the basal lamina was located adjacent to the surface of the ovigerous cords (G, arrows). Cells in the ovigerous cords and stroma were proliferating (A, Ki67 positive, green) and cords were still open at the surface (B–F, indicated with star). Stromal cells in the cortex and medulla expressed COUP-TFII (C, red nuclear staining). Stromal areas between ovigerous cords contained capillaries (F, open arrow, endothelial cells stained for von Willebrand factor, red) and their sub-endothelial basal laminas were composed of, amongst other things, perlecan (F, green) and components of laminin 111 (H, red). The medulla contained arterioles (H, open triangle, green lectin binding to endothelial cells surrounded by extensive basal lamina of smooth muscle cells identified by staining of components of laminin 111), capillaries (filled triangle) and mesonephric-derived tubules or rete ovarii close to the hilus (H, star). Later in development, increasing amounts of stroma led to a breakdown of large ovigerous cords into smaller cords (I, basal laminas stained for components of laminin 111, red) and the development of a basal lamina below layers of GREL cells on the ovarian surface (J, basal laminas stained for components of laminin 111,red). Primordial follicles firstly developed in the inner cortex/medulla region, whereas the outer cortex still contained ovigerous cords [k, basal laminas stained for components of laminin 111 (red)]. In addition to components of laminin 111 (K), the follicular basal lamina contained collagen type IV (L, red) and nidogen 2 (M, red). Nuclei were counterstained with DAPI (blue). Gestational ages: 73 (G), 86 (H), 93 (A), 98 (B–D,F), 120 (E), 127 (I), 144 (J), 148 (K,L) and 171 days (M). Bars: H, K, M = 100 µm; A–F, I, L = 50 µm; J = 25 µm; G = 1 µm.

At early stages the bovine ovary contained GREL cells positive for cytokeratin 19 (Table 3, Fig. 1A), similar to observations in the gonadal blastema of fetal rat ovaries [21]. Interspersed between the GREL cells were PGCs, marked by nuclear expression of octamer-binding transcription factor 3/4 (OCT3/4; POU-domain class 5 transcription factor) (Fig. 1B), which had migrated through the mesonephric stroma and appeared as oogonia close to the surface. The mesonephric surface epithelium and its underlying basal lamina did not extend beyond the ovarian base where it was continuous with the mesonephric stroma (Fig. 1B). More apically in the ovary, basal lamina material was interspersed between cells and was positive for components of laminin 111 (Fig. 1A,B), collagen type IV (Fig. 1C), perlecan (Fig. 1D) and nidogens 1 (Fig. 1E) and 2 (Fig. 1F). Similar laminin deposits have been observed between cells of the gonadal blastema in differentiating rat ovaries [21]. These basal laminas subsequently expanded to form compartments as described below. On one side of the basal lamina were stromal cells connected with and extending from the mesonephric stroma.

#### Establishment of ovigerous cords (70–130 days)

**Figure 3:**
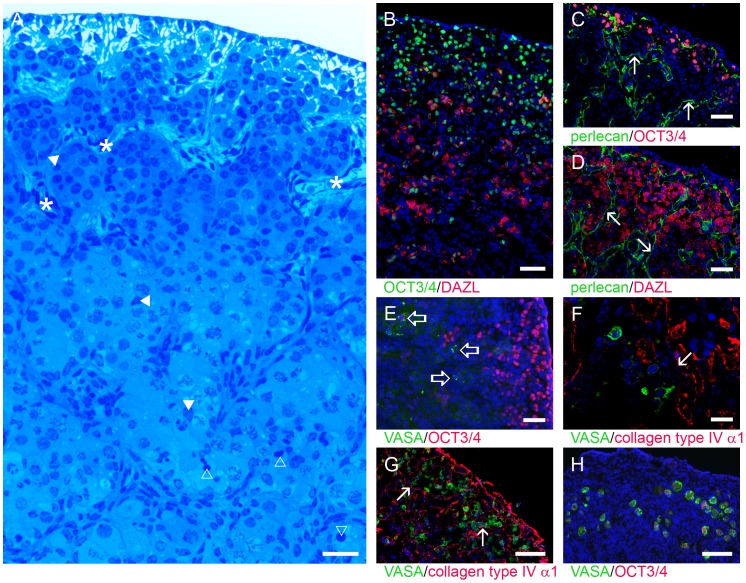
Gradation of germ cell maturation. After migrating into the early-stage ovary, PGCs proliferated as oogonia and together with the GREL cells constituted the ovigerous cords (A, methylene blue-stained semi-thin section, GREL cells marked with filled triangle and capillaries with asterisk). The least mature oogonia were located towards the outer cortex, undergoing mitosis, whilst oogonia in the inner cortex differentiated into oocytes. This zonal differentiation was mirrored in the expression of germ cell markers. Germ cells close to the surface expressed OCT3/4 (B, green; C and E, red), whilst deeper in the ovary they had commenced expressing cytoplasmic DAZL (B and D, red) and later VASA (E, green, open arrows; F, green) with concomitantly reduced OCT3/4 expression. Later in development germ cells in the outer cortex differentiated and expressed VASA (G, green and basal lamina stained for collagen type IV α1). Follicular oocytes expressed VASA (H, green) and OCT3/4 again (H, red). Morphological differences between germ cells and GREL cells were evident; germ cells were large and round with a round nucleus whereas GREL cells were smaller, elongated with an elongated nucleus and a larger nuclear/cytoplasmic ratio. GREL cells were located in the periphery of the ovigerous cords close to the surrounding basal lamina. Occasionally apoptotic germ cells were seen (A, open triangle). Basal laminas (arrows) are indicated by positive staining for nidogen 2 (C, green), perlecan (B and D, green), and collagen type IV α1 (F, red). Nuclei were counterstained with DAPI (blue). Gestational ages: 82 (B), 98 (E), 120 (C,D,F), 132 (G), 134 (A) and 171 days (H). Bars: G, H = 100 µm; B–E = 50 µm; A, F = 25 µm.

Later in development stromal cells from the medulla region of the developing ovary extended toward the surface, infiltrating the areas containing the heterogeneous cell populations of germ and GREL cells. It was the areas of stroma alternating with areas of germ and GREL cells that formed the irregularly-shaped ovigerous cords (Figs. 2A–F, 3A). These have been observed in human fetal ovaries from week 15 onwards [22] and in fetal rat ovaries after day 14.5 [21]. At this stage there was still no distinct surface epithelium with an underlying basal lamina covering the whole ovary and the ovigerous cords were still open to the surface (Figs. 2A–E) as previously observed in sheep [10] and cattle [23].

Based on their differentiation status, germ cells expressed the pluripotency marker OCT3/4 (nuclear staining) and/or DAZL (deleted in azoospermia-like) (cytoplasmic staining). In the outer region of the ovary, germ cells were OCT3/4-positive and DAZL-negative, in the middle region they were both OCT3/4- and DAZL-positive and in the inner region they were OCT3/4-negative and DAZL-positive (Fig. 3B–D). This zonal gradient in germ cell differentiation and expression of markers has also been observed in human fetal ovaries [24], [25]. Around 100 days of fetal development the first VASA (DEAD/H (Asp-Glu-Ala-Asp/His) box polypeptide 4)-positive germ cells (cytoplasmic staining) were detected in the inner cortex/medulla region (Fig. 3E,F), later in development extending to the outer cortex (Fig. 3G). In contrast in human and mouse [26], VASA-positive germ cells could be found earlier in development at the gonadal ridge stage. A few germ cells migrated to the surface of the ovary and then became compartmentalised to the surface of the ovary as the stroma spread laterally below the surface of the ovary (Fig. 3C,D).

**Figure 4:**
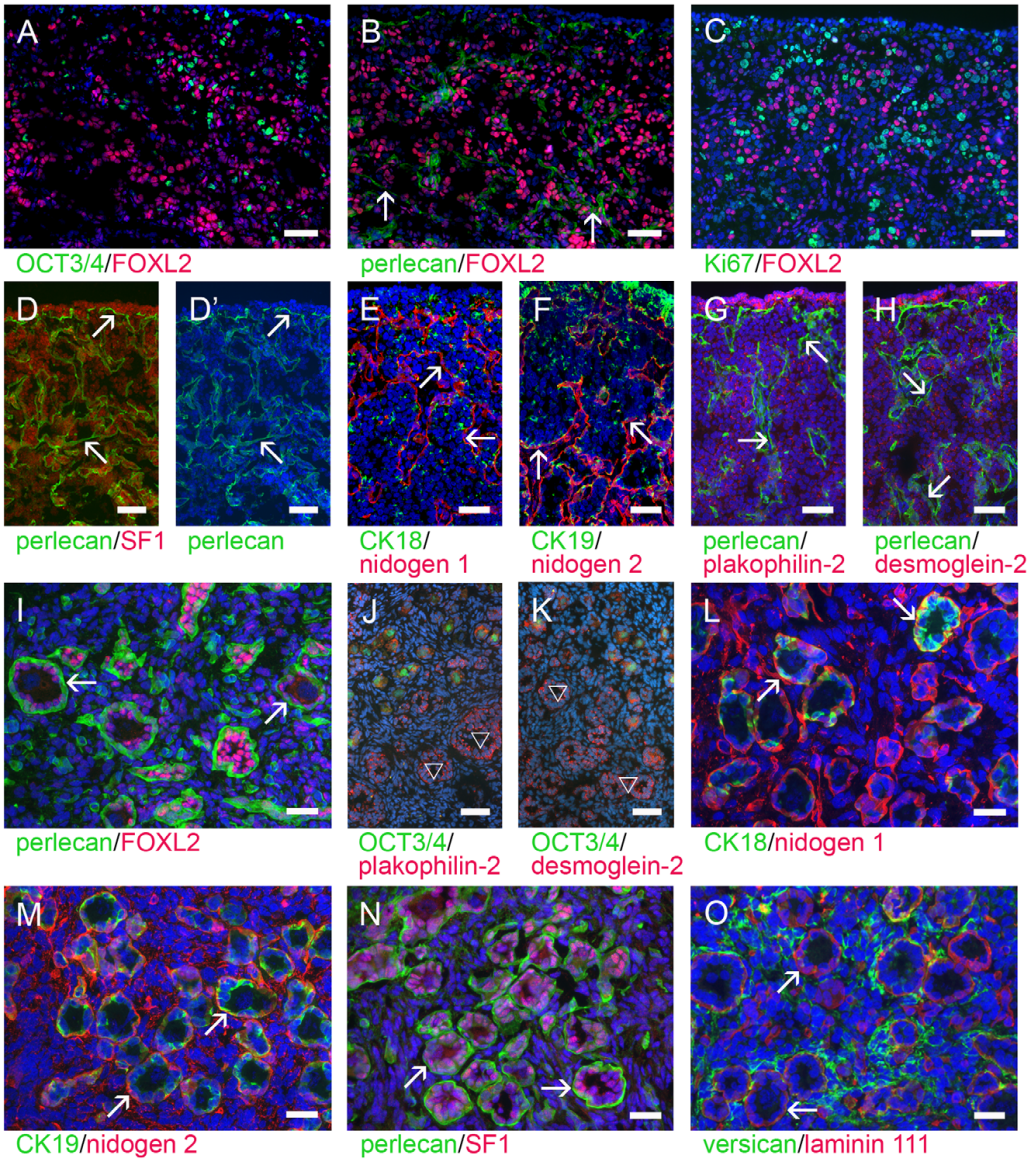
GREL cells in developing follicles. Ovigerous cords were composed of germ cells (A, marked with OCT3/4, green) and GREL cells surrounded by a basal lamina (marked with arrows) composed of, amongst other components, perlecan (B,D,G and H, green) and nidogens 1 (E, red) and 2 (F, red). GREL cells expressed nuclear FOXL2 (A–C, red) and SF1 (D, red), cytoplasmic cytokeratin 18 (E, green) and cytokeratin 19 (F, green) and the desmosomal proteins plakophilin-2 (G, red) and desmoglein-2 (H, red). Cytokeratin 19 as well as plakophilin-2 and desmoglein-2 were more strongly expressed in the cells close to the surface of the ovary. FOXL2 expressing GREL cells (C, red) appeared to be non-proliferating, as there was no co-localization with Ki67-positive cells (C, green). Stromal cells expressed FOXL2 more weakly (A–C, red). The granulosa cells in primordial follicles and activated growing follicles expressed FOXL2 (I, red), plakophilin-2 (J, red), desmoglein-2 (K, red), cytokeratin 18 (L, green), cytokeratin 19 (M, green) and SF1 (N, red). The follicular basal lamina was composed of perlecan (I and N, green), nidogens 1 (L, red) and 2 (M, red), components of laminin 111 (O, red) and collagens type IV and type XVIII (not shown). Follicular oocytes again expressed OCT3/4 (J and K, green). Stromal tissue surrounding follicles contained short fibres of nidogens 1 (L, red) and 2 (M, red) and thick fibres of versican (O, green). Nuclei were counterstained with DAPI (blue). Gestational ages: 86 (E,F), 90 (A–D’,G,H), 171(I–K,M,N), 181 (L) and 193 days (O), respectively. Bars: A–H, J, K = 50 µm; I, L–O = 25 µm.

GREL cells in the ovigerous cords were strongly positive for Forkhead box L2 (FOXL2) (Fig. 4A–C), which has been previously used and described as a marker of granulosa cells in mice [27], [28], [29], goat [30] and human [31]. FOXL2-positive cells were also non-proliferating (Ki67-negative; Fig. 4C); consistent with the observation in fetal mouse ovaries [32]. Additionally, a subset of GREL cells stained positively for cytokeratin 18 (Fig. 4E) and cytokeratin 19 (Fig. 4F), as well as the desmosomal proteins plakophilin-2 (Fig. 4G) and desmoglein-2 (Fig. 4H). Steroidogenic factor 1 (SF1) was expressed in the nuclei of cells of the ovigerous cords (Fig. 4D).

**Figure 5:**
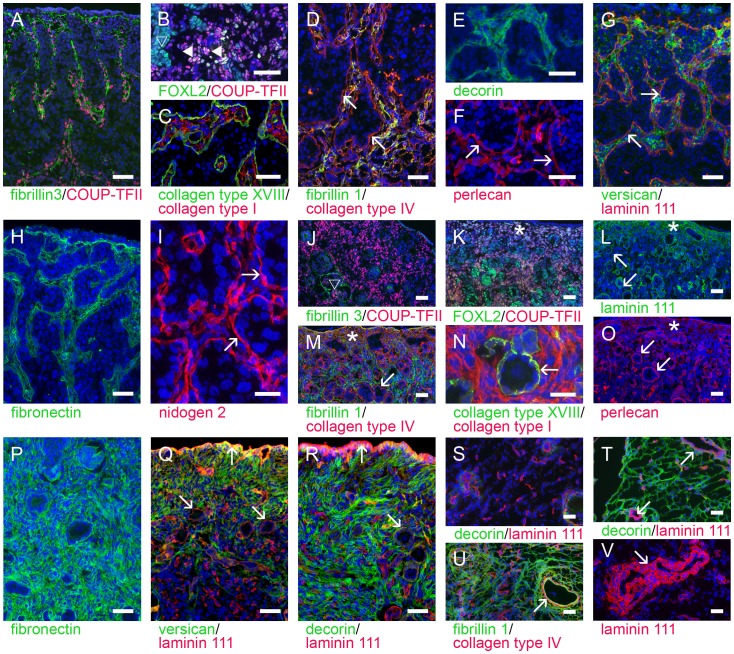
Identification of stroma. The ovigerous cords were separated from the stroma by a basal lamina (marked with arrows), containing amongst other components, collagen type XVIII (C, green), collagen type IV (D, red), perlecan (F, red), components of laminin 111 (G, red) and nidogen 2 (I, red). Stromal cells proliferated and synthesised matrix fibrils containing fibrillin 3 (A, green), collagen type I (C, red), fibrillin 1 (D, green; co-localised yellow with collagen type IV), decorin (E, green) and fibronectin (H, green). Additionally small aggregates of perlecan (F, red) and nidogen 2 (I, red) were observed in the stroma. Versican was localised mainly to the stroma and to a lesser amount to the cytoplasm of ovigerous cord cells in the outer cortex (G, green). Stromal cells expressed nuclear COUP-TFII (A and B, red); but also FOXL2 weakly (B, green, white-yellowish co-localized with COUP-TFII, closed triangle). Conversely FOXL2 was highly expressed in the GREL cells (B, green, open triangle). The tunica albuginea was composed of fibrillin 1 (M, green), collagen type IV (M, red, star) and decorin (R, green) with less laminin (L, green, star and R, red) and perlecan (O, red, star). The stroma of the medulla and cortex contained fibres strongly stained for fibrillin 1 (M, green), collagen type I (N, red), fibronectin (P, green) and decorin (R, green). During development, fibrillin 3 expression declined, showing only remaining fibres around some cell clusters (J, green, open triangle). Versican was restricted to the tunica albuginea and the outer cortex (Q, green) and not expressed in the medulla (S, green). In general the medullar stroma appeared less cellular than the cortical stroma (S-V). Decorin (T, green) and fibrillin 1 (U, green) were localised to the medullar stroma, whereas components of laminin 111 (L and S, Q, R and V, red), collagen type IV (M and U, red), collagen type XVIII (N, green) and perlecan (O, red) were restricted to the basal laminas. Stromal cells in the medulla continued to express COUP-TFII (J and K, red, nuclear) and FOXL2 weakly (K, green, co-localization with COUP-TFII appears red-yellowish), which was mainly expressed in granulosa cells. Nuclei were counterstained with DAPI (blue). Gestational ages: 86 (E), 98 (A–D,F–H), 120 (I), 171 (J–O,U), 183 (P), 190 (T), 229 (Q,R), 236 (S) and 268 days (V). Bars: A–H, J–M, O–V = 50 µm; I, N = 25 µm.

**Figure 6:**
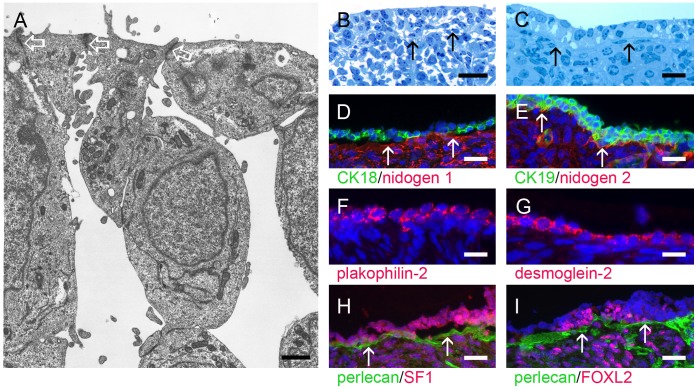
Surface epithelium formation. Early-stage ovaries did not have a specialised surface epithelium under laid by a basal lamina, except at their base where they connected to the mesonephros. However, GREL cells in the most outer layers were connected to each other by adherens junctions (A, electron micrograph, open arrows). During development stromal cells migrated closer to the surface (B, methylene blue-stained semi-thin section, arrows) and expanded laterally over the top of the ovigerous cords until the whole ovarian surface had some layers of GREL cells under laid by a basal lamina and stroma (C, methylene-blue stained semi-thin section, arrows). These GREL cells differentiated into surface epithelium and expressed cytokeratin 18 (D, green) and cytokeratin 19 (E, green), the desmosomal proteins plakophilin-2 (F, red) and desmoglein-2 (G, red) in their cell membranes, and SF1 (H, red) and FOXL2 (I, red). Nidogens 1 (D, red) and 2 (E, red), and perlecan (H and I, green) were localized to the basal lamina (marked with arrows) underlying the ovarian surface epithelium. Nuclei were counterstained with DAPI (blue). Gestational ages: 73 (A), 148 (B), 171 (C), 190 (D), 229 (G,H) and 241 days (E,F). Bars: D–I = 50 µm; B = 25 µm; C = 10 µm; A = 1 µm.

We also observed less intensely stained FOXL2-positive cells in the stroma (around 70% of total stroma cells, Figs. 4A–C, 6B) which is consistent with observations in humans, showing FOXL2 expression in somatic cells surrounding germ cell clusters [33]. Furthermore in mice, FOXL2-expressing cells can give rise to theca cells [34] and theca cells in follicles also express FOXL2 [35]. The stromal cells expressed nuclear chicken ovalbumin upstream promoter-transcription factor (COUP-TFII) (Figs. 2C, 5A,B). COUP-TFII has been shown to be expressed in stroma and especially in theca cells in adult human and mouse ovaries [36], [37] as well as cultured bovine theca cells [38]. No COUP-TFII expression was observed in cells in the ovigerous cords (Figs. 2C, 5A). Most of the stromal cells were SF1-positive, but more weakly than the cells in the ovigerous cord.

The ovigerous cords were separated from the stromal areas by a basal lamina (Fig. 2G), as previously shown in fetal ovaries of cattle [13] and sheep [10]. The basal lamina contained collagen type IV (Fig. 2A), collagen type XVIII (Fig. 2B), nidogens 1 (Fig. 2D) and 2 (Fig. 2E), perlecan (Fig. 2F) and components of laminin 111 (Fig. 2H,I,J). The stroma between ovigerous cords contained capillaries (Fig. 2F). The capillaries were surrounded by a sub-endothelial basal lamina containing components of laminin 111 (Fig. 2H), collagens type IV and XVIII (Table 3), perlecan (Fig. 2F), and nidogens 1 and 2. Stroma was positive for collagens type I (Fig. 5C) and IV (Fig. 5D), fibrillins 1 (Fig. 5D) and 3 (Fig. 5A), as observed previously in bovine and human [2], fibronectin (Fig. 5H), versican (Fig. 5G), decorin (Fig. 5E) and weakly positive for components of laminin 111 (Fig. 5G), perlecan (Fig. 5F) and nidogens 1 (Table 3) and 2 (Fig. 5I).

In contrast to the cortex, which was characterized by the ovigerous cords, the medulla predominantly contained stroma, vasculature and rete ovarii in the hilus (Fig. 2H). The medullar stromal matrix was composed of the same molecules as the stroma between the ovigerous cords (Figs. 2I, 5D,H). The sub-endothelial basal lamina contained components of laminin 111 (Fig. 2H), collagens type IV and XVIII, perlecan and nidogens 1 and 2 (Table 3). The basal laminas of smooth muscle cells of arterioles were positive for components of laminin 111 (Fig. 2H), collagen type XVIII, perlecan and nidogens 1 and 2 (Table 3).

As the stroma increased during ovarian development the large ovigerous cords became segmented into smaller clusters of cells, commencing in the regions closest to the medulla (Fig. 2I). Stromal cells were also now apparent beneath the outer most layers of cells on the surface forming a stromal layer separating cells on the surface from the ovigerous cords (Fig. 2J,K). A basal lamina underlying the surface epithelium was thus formed, containing components of laminin 111 (Figs. 2J,K, 5L,Q,R), collagen type IV (Fig. 5M), collagen type XVIII (Table 3), perlecan (Figs. 5O, 6H,I) and nidogens 1 (Fig. 6D) and 2 (Fig. 6E). Ovarian surface epithelial cells were positive for cytokeratin 18 (Fig. 6D) and 19 (Fig. 6E) and SF1 (Fig. 6H), as has been shown for neonatal and adult ovine ovaries [39] and adult human ovaries [40]. Desmosomal proteins plakophilin-2 (Fig. 6F) and desmoglein-2 (Fig. 6G) were localized to the cell surface. Plakophilin was highly expressed in human ovarian surface epithelium [18] and desmoplakin, another component of desmosomes, has been detected in human, pig and rat ovarian surface epithelial cells, which were also positive for cytokeratins [41]. Generally ovarian surface epithelial cells were negative for FOXL2 (Fig. 5K), but sporadically FOXL2-positive cells were visible (Fig. 6I).

#### Follicle formation (>130 days)

Later in the developing ovary the breakdown of the ovigerous cords into smaller groups of germ and GREL cells resulted in the formation of primordial follicles containing an oocyte surrounded by flattened granulosa cells separated from surrounding tissue by a basal lamina. The primordial follicular basal lamina contained the same components as the basal lamina surrounding the ovigerous cords, with components of laminin 111 (Figs. 2K, 4O, 5L,Q,R), perlecan (Figs. 4I,N, 5O), collagen type IV (Figs. 2L, 5M), collagen type XVIII (Fig. 5N) and nidogens 1 (Fig. 4L) and 2 (Figs. 2M, 4M). The first follicles were formed close to the medulla while the cortex still contained shorter ovigerous cords (Fig. 2K). Later primordial follicles were formed closer to the surface. This gradual progression of follicle formation extending from the medulla to the surface has been observed in fetal ovaries of human [22], cattle [23], [42] and sheep [10], [43] and in postnatal rat ovaries [44].

The oocytes of primordial and primary follicles (where granulosa cells were cuboidal and replicating) were VASA positive (Fig. 3F) and some also OCT3/4 positive (Fig. 3F). In mice, it has been shown that OCT4 is down-regulated when oogonia enter meiosis, but is then up-regulated after the final stages of meiotic prophase I [45]. On the other hand in human fetal ovaries it was observed that VASA was expressed in oogonia and oocytes, whereas OCT3/4 was not detectable in oocytes at any follicle stage [46].

Granulosa cells of primordial and growing follicles expressed FOXL2 (Fig. 4I), cytokeratin 18 (Fig. 4L), cytokeratin 19 (Fig. 4M) and SF1 (Fig. 4N). It has been shown in human and rat ovaries that the expression of cytokeratins appears to decrease during follicle maturation, whereas there was no cytokeratin expression in pig granulosa cells [21], [41]. Granulosa cells also expressed plakophilin-2 (Fig. 4J) and desmoglein-2 (Fig. 4K).

At the stage when preantral and later antral follicles appeared, the ovary underwent further changes: the cells on the ovarian surface became single-layered and the stroma underlying the surface thickened, forming the tunica albuginea (Fig. 5M,K,L,O). The basal laminas of follicles, capillaries and ovarian surface epithelium contained components of laminin 111 (Fig. 5L,Q,R), collagens type IV (Fig. 5M) and XVIII (Fig. 5N), perlecan (Fig. 5O) and nidogens 1 (Fig. 4L) and 2 (Fig. 4M). Smooth muscle cells around arterioles in medulla and cortex expressed components of laminin 111 (Fig. 5T,V), perlecan, collagens type IV and XVIII, and nidogens 1 and 2 which are also found in the tunica albuginea (Fig. 5L,M,O) as well as versican (Fig. 5Q), decorin (Fig. 5R), fibronectin, collagen type I (Table 3). The medullar stroma formed fibres of decorin (Fig. 5T), fibronectin (Table 3), fibrillin 1 (Fig. 5U) and collagen type I (Fig. 5N). Stromal cells around follicles still expressed COUP-TFII (Fig. 5J,K) and to some extent FOXL2 (Fig. 5K). There was no expression of COUP-TFII in granulosa cells of preantral or antral follicles as observed in human adult ovaries [36].

## Discussion

**Figure 7:**
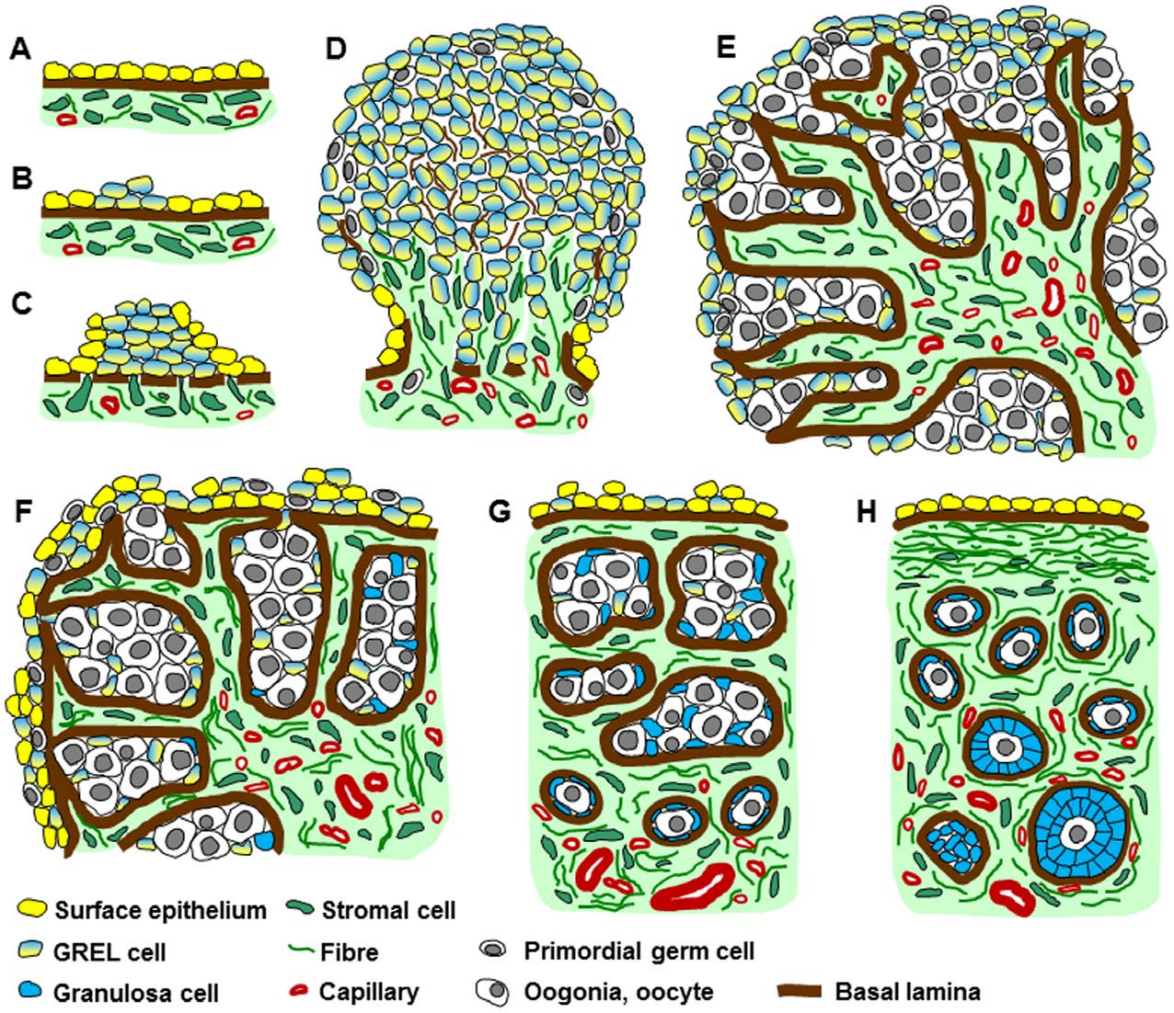
Schematic diagram of ovarian development. (A) The development of the ovary commences at the mesonephric surface epithelium (yellow cells) in the location of the future gonadal ridge. (B) Some mesonephric surface epithelial cells change phenotype into GREL cells (yellow-blue cells). (C) The GREL cells proliferate and the basal lamina underlying the mesonephric surface epithelium breaks down allowing stromal cells (green) to penetrate into the gonadal ridge. (D) GREL cells continue to proliferate and PGCs (grey) migrate into the ridge between the GREL cells. Mesonephric stroma including vasculature (red) continues to penetrate and expand in the ovary. (E) Oogonia proliferate and stroma penetrates further towards the ovarian surface enclosing oogonia and GREL cells into ovigerous cords. The cords are surrounded by a basal lamina at their interface with stroma, but are open to the ovarian surface. Stromal areas including those between the ovigerous cords contain capillaries. (F) A compartmentalization into cortex and medulla becomes obvious. The cortex is characterised by alternating areas of ovigerous cords and stroma, whereas the medulla is formed by stromal cells, vasculature and tubules originating from the mesonephros (rete ovarii). Once stroma penetrates below the cells on the surface it spreads laterally. The GREL cells at the surface are then aligned by a basal lamina at their interface with the stroma and begin to differentiate into typical ovarian surface epithelium (yellow cells). Some germ cells at the surface are also compartmentalized to the surface as stroma expands below it. (G) Ovigerous cords are partitioned into smaller cords and eventually into follicles. These contain GREL cells that form granulosa cells (blue cells) and oogonia that form oocytes. The first primordial follicles appear in the inner cortex-medulla region, surrounded by a basal lamina. A now fully intact basal lamina underlies multiple layers of surface epithelial cells. (H) At the final stage the surface epithelium becomes mostly single-layered and a tunica albuginea, densely packed with fibres, develops from the stroma below the surface epithelial basal lamina. Some primordial follicles become activated and commence development into primary and preantral follicles.

We have been able to make our seminal discoveries on the development of the ovary and formation of follicles using the following strategies. We identified markers of GREL cells following cell culture, clonal isolation and microarray analysis. By immunohistochemical staining for extracellular matrix components (collagen I, decorin, fibronectin and fibrillins 1 and 3), we were able to identify and track the development of stroma. We also identified basal lamina components (collagens type IV and XVIII, laminin, nidogens, perlecan and electron microscopy), and discovered a basal lamina at the interfaces of the ovarian stroma and all other compartments. Thus we could accurately delineate the ovigerous cords, follicles and surface epithelium as they developed. Our studies enabled us to propose a novel model of the formation of the ovary and follicles as illustrated in Fig. 7.

The gonadal ridge forms on the ventral side of the mesonephros; elongated in an anterior/posterior direction which then develops into the ovarian primordium. A new observation is that when it is first formed it does not have a defined surface epithelium except at the base where it arises and protrudes from the mesonephros. A true epithelium would have a sub-epithelial basal lamina and an epithelial-stromal interface which the ovarian primordium/gonadal ridge does not have. Instead the ovarian primordium/gonadal ridge is composed of a cluster of GREL cells which developed either from the surface epithelium of the mesonephros or less likely from the stroma just below the surface. Our evidence suggests the former since the GREL cells express epithelial markers (cytokeratin 18, cytokeratin 19) and have cell-cell junctions (plakophilin-2, desmoglein-2). Additionally the GREL cells are clearly not the same as stromal cells as seen in the mesonephros, nor do they express stromal matrices (collagen type I, decorin, fibronectin or fibrillins 1 or 3). If GREL cells developed from the stroma below the surface epithelium of the mesonephros then it would be expected that a surface epithelium would continue to cover the ovarian primordium as it grows, which is not the case. The partition formed by a basal lamina (containing components of laminin 111, collagen type IV, collagen type XVIII, perlecan and both nidogens 1 and 2, and also observed by electron microscopy) at the interface of GREL cells and the stroma as it penetrates into the gonadal ridge, also indicates that the GREL cells are not derived from the mesonephric stroma but instead from the mesonephric surface epithelium. Without strong evidence others have also come to similar conclusions [4], [47].

During the formation of the ovary, the stroma from the mesonephros penetrates into the gonadal ridge/ovarian primordium composed of GREL cells and PGCs. This process creates areas of stroma alternating with areas of GREL cells/germ cells and hence produces the ovigerous cords which are composed of GREL cells and germ cells ‘open’ to the surface. The penetrating stroma has been observed previously and described as ‘cell streams’ [11]. A new observation is that at all times there is continuous basal lamina between the stroma and the ovigerous cords, between the stroma and follicles and between the stroma and the surface epithelium. The composition of the basal laminas in these locations is identical and contains components of laminin 111, collagens type IV and XVIII, perlecan and both nidogens 1 and 2. This supports the notion that cords, follicles and surface epithelium are formed or compartmentalized by the penetrating stroma, which therefore must be pivotal in their formation.

A further new observation is that when the ovarian primordium first develops it lacks a mature surface epithelium. The cells at the surface are similar to the GREL cells deeper in the ovary and all express epithelial markers (cytokeratin 18, cytokeratin 19) and have cell-cell junctions (plakophilin-2, desmoglein-2). This expression pattern is not the same as in the epithelium that covers the mesonephros and continues only onto the base of the ovary. At these early fetal stages there is no epithelial-stromal interface nor is there a basal lamina underlying superficial GREL cells as seen with a mature epithelium. It is only at a later stage of development when the stroma has penetrated the ovary and expanded laterally underneath the superficial GREL cells that the superficial GREL cells take on the appearance of a mature surface epithelium with a sub-epithelial basal lamina and an epithelial-stromal interface and up regulation of epithelial markers occurs. An additional important consideration of this process is that the surface epithelium of the ovary has a developmental pathway different to the mesothelial cells lining the other peritoneal organs and the peritoneal wall.

The stroma also continues to penetrate the ovigerous cords, partitioning them into smaller groups of germ cells and GREL cells. This process commences closest to the medulla of the ovary where the GREL cells/germ cells first encounter the penetrating stroma and where the germ cells mature into oocytes first, as observed by expression of germ cell markers temporally from Oct3/4, DAZL and then to VASA. Eventually the GREL cells/germ cells are partitioned into groups of cells the size of primordial follicles. Since there is a basal lamina at the interface of the penetrating stroma and the GREL cells, the GREL cells are the only somatic cells inside the basal lamina and must therefore be the precursor of the granulosa cells of follicles.

The presence of a basal lamina at the interface of stroma and the cords initially and later in development at the interfaces with follicles and the surface epithelium suggests that stroma remains physically separated from the GREL cells or their derivates at all stages of ovarian development. The implications for cell lineages are obvious. The surface epithelium and granulosa cells must both be derived from GREL cells. The granulosa cells and the surface epithelium, respectively, have either the oocyte or the abdominal cavity located on their apical sides. It would be predicted that the oocyte influences the GREL cells to form granulosa cells and indeed growth factors from oocytes have been shown to regulate granulosa cells [48], [49].

Since the penetrating stroma has a basal lamina at its leading edge this reinforces previous suggestions that the tunica albuginea and cortical stroma in adult ovaries are of another lineage derived from and continuous with mesonephric stroma. Since the tunica is adjacent to the surface epithelium it could be that the surface epithelial cells influence the behaviour of the adjacent stromal cells to develop into the fibrous tunica. The other stromal compartments in adult ovaries are the specialized theca interna and externa layers that arise adjacent to and on the stromal side of the follicular basal lamina in developing antral follicles. The theca interna synthesizes androgen precursors for estradiol synthesis, whereas the role of the theca externa has not been identified but it may be important for follicular fluid expulsion during ovulation. The current study does not identify the origin of thecal cells but suggests that their origins have nothing in common with either granulosa cells or surface epithelium, since these cells are located on the other side of a basal lamina at all stages. More likely the thecal cells arise from stem cells [50] within the stroma and indeed a potential stem cell niche in the theca has recently been identified [51].

The adult bovine ovary is composed of a cortex and a medulla like many other species including the human [12] and it appears that the cortex is largely the area initially occupied by the ovigerous cords separated from each other by the penetrating stroma. The area below the cortex, the medulla of the adult ovary, is largely residual mesonephric stroma containing residual mesonephric tubules (rete ovarii) and vasculature. When the stroma penetrates into the gonadal ridge/ovarian primordium, it contains endothelial cells assembled into mature capillaries surrounded by a sub-endothelial basal lamina. Thus this capillary network of the ovarian cortex is not likely formed by the vascularisation process but rather by sprouting or splitting forms of angiogenesis [52] allowing expansion of the existing capillary network derived directly from vasculature in the mesonephros.

Each tubule of the rete ovarii is surrounded by a basal lamina (containing components of laminin 111, collagen type IV, perlecan and nidogens 1 and 2) and the tubules are not connected to any ductal system, unlike in the testis [53]. Whilst some earlier studies suggested they could be a source of granulosa cells or affect follicle development we found no morphological role in the fetal ovarian cortex where the follicles are formed, and there was no evidence that they form the granulosa cells of follicles. However, our studies do not rule out roles for rete ovarii in adult ovaries or indeed any paracrine roles either in fetal or adult ovaries.

Like others [54], [55], [56], [57] we also observed some germ cells at the surface of the ovarian primordium. Importantly we observed that these cells became localized to the surface of the ovary by means of the stroma spreading laterally below the surface of the ovary which at that stage contains GREL cells and germ cells. The fate of all of these germ cells is not known. Some are lost from the surface of the ovary into the peri-ovarian space as reported previously in humans and mice [54], [55], [56], [57], or they could subsequently undergo cell death. It is speculated that if these cells survive at the surface, they could be the source of the germ-line stem cells isolated from the surface or the outer cortex of the ovary [58], [59].

We believe our study also highlights an under appreciated role of the stroma in the formation of ovigerous cords, surface epithelium and follicles. Additionally the stroma is important because as it penetrates what will become the cortex of the ovary, it contains a vascular capillary bed and thereby provides a blood supply to the cortex. Aberrant stromal activity may also be important in human conditions such as PCOS. It is well known that PCOS ovaries have increased numbers of antral follicles but less well appreciated that they also have substantially more tunica albuginea containing more collagen and they also have increased thicknesses of the cortical and sub-cortical stroma [60]. This fact has been known since the first report of PCOS [61], however, these features of PCOS ovaries had not received much attention until recently when it was discovered that the fibrillin 3 gene, located in the a genomic region associated with PCOS [62], is expressed in the penetrating stroma in ovaries in the first trimester [2]. In that study it was concluded that ‘*since fibrillins are stromal matrices and since the ovarian stromal compartments are altered in women with PCOS, fibrillin 3 expression in the developing fetal ovary, via the activity of TGFβ to regulate stroma formation and function, could predispose an individual to PCOS in later life*’ [2]. Clearly the role of ovarian stoma warrants further investigation.

In summary, we have identified how the somatic cells of the mesonephros contribute to the formation of the ovary and follicles and how they produce the ovigerous cords, ovarian cortex, medulla, tunica albuginea and the granulosa cells and surface epithelium. We have also identified the cell lineages and hence the cell fate decisions needed for maturation of different somatic cells in the adult ovary. These findings are of major importance for our understanding of the formation of the ovary and follicles, of key diseases or disorders of the ovary and, given the common origins with the testis and adrenal cortex, potentially to these organs as well.

## Supporting Information

Figure S1 **Three different cell types were observed in cultures of fetal ovaries.** A spindle-shaped fibroblastic cell type (A), a granulosa cell type (B) and polygonal cells (C,D) were observed. The granulosa cell type (B) was flattened with no visible nucleus and abutted each other closely without gaps between the cells. These cells resembled granulosa cells cultured in a medium containing fetal calf serum and were only observed in ovaries which contained follicles (>125 days). In mixed cell cultures (E) the polygonal cell type formed characteristic clusters distinctly separated from the other two cell types. We focused on these polygonal cells, which we later identified as GREL cells, as they appeared to be novel and were not fibroblasts, granulosa cells or surface epithelial cells. They were neither flattened nor tightly packed. They contacted each other at irregular intervals, possibly indicating the presence of focal cell-cell junctions and were individually raised on the surface of the dish with visible nuclei. Gestational ages were 86 (A), 127 (E), 134 (B), 238 (C) and 241 days (D). Bars: A–E = 100 µm.
(TIF)
