# Supplementary figures and images for: A New Model of Development of the Mammalian Ovary and Follicles
Source: PLoS One. 2013 Feb 7;8(2):e55578. doi: 10.1371/journal.pone.0055578 (PMC3567121; doi:10.1371/journal.pone.0055578)

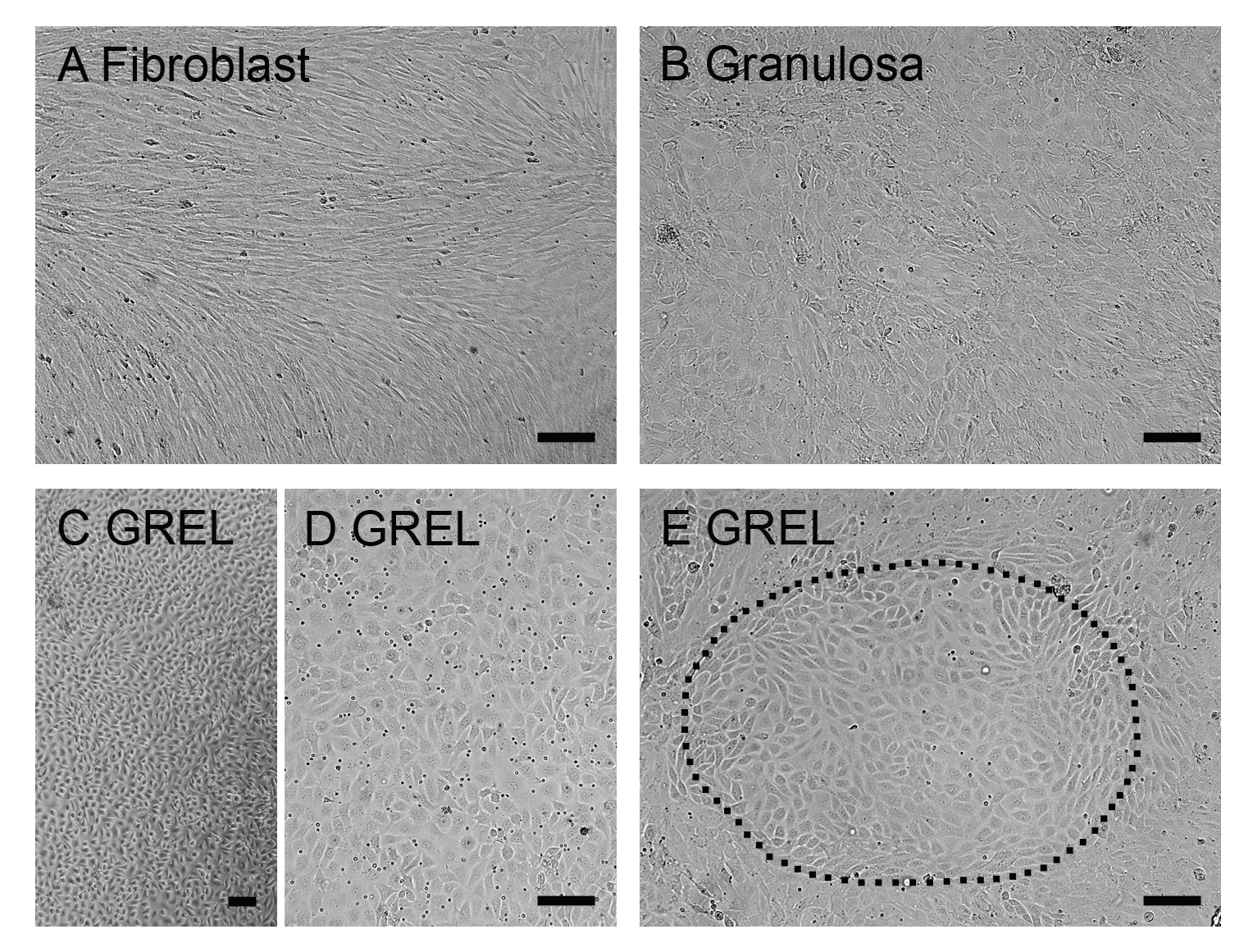

Supplement: Figure S1 — Three different cell types were observed in cultures of fetal ovaries. A spindle-shaped fibroblastic cell type (A), a granulosa cell type (B) and polygonal cells (C,D) were observed. The granulosa cell type (B) was flattened with no visible nucleus and abutted each other closely without gaps between the cells. These cells resembled granulosa cells cultured in a medium containing fetal calf serum and were only observed in ovaries which contained follicles (>125 days). In mixed cell cultures (E) the polygonal cell type formed characteristic clusters distinctly separated from the other two cell types. We focused on these polygonal cells, which we later identified as GREL cells, as they appeared to be novel and were not fibroblasts, granulosa cells or surface epithelial cells. They were neither flattened nor tightly packed. They contacted each other at irregular intervals, possibly indicating the presence of focal cell-cell junctions and were individually raised on the surface of the dish with visible nuclei. Gestational ages were 86 (A), 127 (E), 134 (B), 238 (C) and 241 days (D). Bars: A–E = 100 µm. (TIF) [file pone.0055578.s001.tif]
